# Report of Cases of Typhoid Fever and Typhoid Pneumonia, Treated Principally with Veratrum Viride

**Published:** 1852-05

**Authors:** W. J. Summer

**Affiliations:** Lexington, S. C.


### SELECTIONS.

From the Southern Med. and Surg. Journal.

*Report of Cases of Typhoid Fever and Typhoid Pneumonia, treated principally with Veratrum Viride.* By W. J. SUMMER, M. D., of Lexington, S. C.

SINCE the publication of Dr. Norwood's articles on Veratrum Viride, I have extensively employed it in the treatment of Typhoid fever and Pneumonia, and with the most gratifying success. I would, therefore, most cordially adduce my experience in support of the remedy, as being at once safe and efficient. Before I became acquainted with this valuable agent, I regarded the treatment of a severe case of Typhoid fever with something like dread, as to its results, but now I am less concerned about my cases of this disease, than of other affections generally deemed more trivial. A brief notice of a few cases treated with this remedy, will not, I hope, prove unacceptable to the profession, and will, perhaps, induce others to make trial of this valuable agent.

CASE I. Rebecca, aged 8, daughter of Mr. F. I was called to see her on the 5th October last. She had been suffering under the premonitory symptoms of Typhoid fever for four or five days, and the disease was now fully formed. There was headache; hot, dry skin; small and frequent pulse; tongue coated with a whitish fur, with tips and edges red and rather dry; loss of appetite, amounting to a disgust for food. There was tenderness of the abdomen in the right iliac region, pressure over which produced the gurgling noise so characteristic of this affection—no diarrhœa. I prescribed pil. hydrarg. gr. v., to be followed, if it did not act on the bowels, by castor oil. Scarified cups were applied to the abdomen—pepper poultices to be used subsequently. After the bowels were cleared, neutral mixt. with a minute quantity of tartar emetic. (I had no veratrum viride with me.)

Oct. 6th. Called and found my patient in pretty much the same condition: had two evacuations from the bowels without taking oil; headache somewhat relieved; abdomen still a little tender; pulse 130. Discontinued previous treatment, and ordered tinct. verat. vir., to be given in doses of three drops every three hours, and if nausea, emesis, or a reduction of the pulse did not take place after giving three or four doses, to increase the dose one drop each time. Pepper mush to be kept constantly applied to the abdomen.

7th. Called eighteen hours afterwards. Patient had vomited once, a short time previous; skin cool; pulse reduced to 80: the vomited matters consisted of a quantity of mucus and watery fluid. The dose of tinct. veratrum had been increased to six drops. Directed it to be given in doses of four drops every four hours. At my visit next day, the patient seemed entirely free from fever—pulse 72, soft and full. Bowels had acted twice since previous visit, dejections resembled what had been vomited. The dose was now reduced to three drops, repeated at intervals of three or four hours and continued for several days, for fear of a return of the fever, after which it was discontinued. The patient convalesced rapidly.

Case II. Henry, aged 10, brother to the above. Called 20th Oct. Found this patient with symptoms very much resembling the first, though more severe, particularly the headache. The parents had administered a dose of epsom salts, the bowels having been constipated. The salts, however, acted drastically, and diarrhœa was subsequently very obstinate. There was considerable tenderness in the right iliac region, and gurgling on pressure. Cups were applied to the temples and abdomen, followed by poultices to the latter; hyd. c. creta et pulv. Dov., in small doses, for the diarrhœa, and the patient at once put upon the use of tinct. veratrum, in small doses, gradually increased. Gum-water was the only article of drink or food allowed.

Oct. 21st. Patient complains less of headache; pulse reduced from 130 to 80; has vomited once or twice without much nausea; diarrhœa still persistent, yet the discharges are not so watery. Continue the same prescription—the veratrum in slightly diminished quantity.

22d. Diarrhœa rather worse, there being frequent watery discharges, telling rapidly on the strength of the patient; tongue dry and red, but the skin cool, and pulse only 70; no nausea. The family were averse to using the syringe, or I should have directed opiate enemata; as it was, I had recourse to acet. plumbi combined with pulv. Dov., a dose of which was given every two hours; veratrum to be continued in small doses.

23d. Patient is better to-day. Bowels have acted but three or four times in the last twenty-four hours; tongue less red and dry; pulse still about 70, full and soft; skin cool and moist; no tenderness of the abdomen, though it is still a little tympanitic. Continued same treatment.

24th. Better in all respects; diarrhœa is sufficiently checked; tongue is becoming moist and clean; abdomen more natural; pulse and skin about natural. Discontinued my visits. Next day, the pulse showing a disposition to rise; the father called on me again, and not being well enough to ride, I gave him some of the tinct. veratrum, of which he gave small doses for a few days, with the same happy results. Recovery was slow but steady and perfect.

Case III. A negro girl, aged 7, the property of Mr. H——. Was called to her on the 6th Dec. She had been ill for more than a week previous, and the disease was now fully formed, and unusually severe. The pulse was feeble and very frequent, amounting to 140; the tongue was quite dry and red; the abdomen tender and very much distended; diarrhæa also was present, there being from six to ten discharges daily of a watery fluid, resembling new cider. She was more or less affected with delirium, particularly at night. Scarified cups were applied to the abdomen, followed by poultices; small doses of acet. plumbi with Dover's powder were given every two hours, and tinct. verat. vir. every four hours, commencing with three drops and increasing one drop at each dose until its ordinary effects were obtained.

7th. Patient has less fever this morning; pulse 110; diarrhæa almost as bad as ever; patient rested better through the night, though there was slight delirium. Ordered in addition to the lead and Dover's powder, laudanum injections, and applied a large blister to the abdomen. The veratrum to be continued in full doses.

8th. No fever to-day; pulse 80, full and soft. Patient this morning passed about eight ounces of nearly pure blood from the bowels—blister drew well and the abdominal distention is diminished. Directed acetate lead and opium in large doses, to be repeated as often as could be well borne—veratrum to be continued in diminished doses.

9th. Patient is much better: no hemorrhage from the bowels nor watery stools; pulse 75 and natural; tongue is becoming moist and clean. Left off all medicine except veratrum, which was still to be given in very small doses.

13th. Saw this patient, and she seemed so much improved that I ceased visiting her. Directed the owner to watch her for a day or two, and if she took fever again, to resume the use of veratrum and send for me.

16th. Was summoned in haste to this patient. She had, a few hours before, been attacked with severe pain in the lower part of the abdomen, which rapidly spread over its whole extent—the abdomen was so tender as not to bear the weight of the bed-clothes without pain. The pulse was a mere thread, and so frequent as scarcely to be counted; respiration short and hurried; stomach very irritable—in short, she had all the symptoms of peritonitis, and caused, undoubtedly, by perforation of the intestines, allowing the contents to escape into the cavity of the abdomen.

I attempted the administration of large doses of opium, hoping that under its influence, in connection with perfect rest and the avoidance of all substances internally, which could in any way disturb the bowels, the system might be supported until adhesive inflammation might possibly unite the perforated intestine to the adjacent parts. But such was the irritability of the stomach, that nothing could be retained, and reluctantly I had to abandon the case as utterly hopeless. She died 18 hours after the commencement of the symptoms of peritoneal inflammation.

I regret exceedingly that I had not the privilege or time to make a post-mortem examination; yet I am confident that the disease was, in the first place, typhoid fever, and that the fatal termination was due to perforation of the intestines, followed by general peritonitis. Equally certain am I, also, that had not perforation of the intestine occurred, this patient would have recovered.

Case IV. Mr. S., aged 30, and of delicate constitution. I was called to him on the 4th Jan. last—he had been in bed for four or five days. I found him with the usual symptoms of Typhoid fever, in addition to which he had pain in the left side, a distressing cough, and was expectorating the rust-colored sputa, as characteristic of Pneumonia. On examining his chest, I detected inflammation involving the lower half of the left lung, and, seemingly, verging into the second stage, or that of real hepatization. His tongue was covered with a whitish fur, with tip and edges red and dry; bowels acted about twice a day, without medicine; abdomen a little full, though nearly natural; no appetite whatever. Pulse 128, and without much strength; respiration hurried and laborious.

He was already much prostrated, so I contented myself with the abstraction of a couple of ounces of blood from the side by cupping. I at once put him on the use of tinct. veratrum viride, in doses of eight drops every three hours, gradually increasing the dose until nausea, vomiting, or a reduction of the pulse was induced. Not feeling satisfied to trust the life of my patient to this remedy alone, (I had not used it in such cases,) I desired to employ in conjunction with it, an alterative mercurial course; but no reasoning or persuasive power of mine could induce him to give his consent. He had once been under the specific influence of mercury, and now declared that he would take no more of the subtle mineral.

5th. Patient seems in much the same condition, with the exception of fever. On increasing the veratrum to twelve drops, slight vomiting, without (as the patient said) nausea, was produced, and the pulse came down to 90, at which I found it. Has still some pain in the side, particularly on coughing; expectoration free, and redder and less viscid than in sthenic pneumonia. Applied a blister, 6 by 8 inches, over the inflamed lung; directed veratrum to be given, in full doses, until the pulse was reduced to 70, then diminished one-half, and continued.

6th. Better to-day; veratrum is well borne, has reduced the pulse to 68. Respiration slow and easy; cough less troublesome, and expectorates freely; no pain; blister has drawn well; tongue tremulous and pointed, but moister; bowels move about twice daily; abdomen slightly distended. Veratrum to be continued in doses sufficient to maintain the reduction of the pulse, and along with it, small and frequent doses of the decoction of polygala senega.

7th. Patient still better; feels stronger since taking the seneka. Pulse rather below 70, and strong enough; respiration good, though, of course, a little hurried. Continue same treatment.

9th. Patient still improving; has gained a little more strength; takes light nourishment with some relish. Blister is healing, and the solidified portion of lung is becoming permeable to air, though slowly; the cough is better, and expectoration diminished and natural. Still to take small doses of veratrum, and sulph. quinine grs. ij. thrice daily, as a tonic.

11th. Called and found the patient doing well in every respect, except that his bowels were much too irritable, there having been three or four watery discharges in the last twenty-four hours. Prescribed a combination of acid. sulph. aromat. with tinct. opii, in sufficient quantities to check the diarrhæa; the tinct. opii, then to be continued or omitted, as should be necessary; the acid to be taken freely as a tonic. The patient now improved, and has recovered without farther treatment.

One or two remarks, and I have done. It will be seen that I have not depended on veratrum viride alone, in the treatment of the cases in which I was employed, but have called into requisition other agents of acknowledged therapeutic value, and, as I believe, with better effect than could be obtained from its single action. At the same time, I am confident that without the use of this extraordinary medicine, I should, within the last few months, have lost many patients by Typhoid fever and Pneumonia—indeed, since I have learned its valuable properties, I would not know how to dispense with it. About a year ago I had a case of Typhoid Pneumonia, similar to the one above stated, which, in spite of my best directed efforts ran on to a fatal termination. Had I, at that time, been acquainted with the properties of this invaluable medicine, I have not a doubt but I could have saved this patient. Something was wanted to control the excited circulation, which was beyond my reach. Although it is not the only medicine to be depended on, it is certainly the chief one. I have used the remedy in at least a dozen cases of Pneumonia, after depletion, when that was indicated, and with a success beyond any thing I ever anticipated. With the exception of the case reported in this communication, (Case iii.) I have not lost a case in which I have used the remedy. In a general way, I have not found the medicine act harshly or disagreeably,—it is true, it sometimes makes the patient deathly sick, but its unpleasant effects are easily obviated, and after the system is fully under its influence, its unpleasant effects usually soon pass off. It is applicable to many diseases, but is particularly suited to Pneumonia. By reducing the circulation, it very materially lessens the amount of labor thrown upon the lungs; a circumstance greatly to be desired, when we remember that well established principle—an inflamed organ must have rest. I have found it to possess all the powers and properties ascribed to it by Dr. Norwood, yet I have usually found it necessary to continue it a day or two longer than he directs. I use the saturated tincture of the root.

